# Experiences and perceptions of coercive practices in mental health care among service users in Nigeria: a qualitative study

**DOI:** 10.1186/s13033-022-00565-4

**Published:** 2022-11-23

**Authors:** Deborah Oyine Aluh, Olaniyi Ayilara, Justus Uchenna Onu, Ugnė Grigaitė, Barbara Pedrosa, Margarida Santos-Dias, Graça Cardoso, José Miguel Caldas-de-Almeida

**Affiliations:** 1 Comprehensive Health Research Centre (CHRC), Nova Medical School, Nova University of Lisbon, Rua Do Instituto Bacteriológico nº5, 1150-082 Lisbon, Portugal; 2 Lisbon Institute of Global Mental Health, Lisbon, Portugal; 3 Department of Clinical Pharmacy and Pharmacy Management, University of Nigeria Nsukka, Nsukka, Enugu State, Nigeria; 4 Department of Clinical Services, Federal Neuropsychiatric Hospital, Uselu, Edo State Nigeria; 5 Department of Mental Health, Faculty of Medicine, Nnamdi Azikiwe University, Nnewi Campus, Awka, Anambra State Nigeria

**Keywords:** Coercion, Restraint, Qualitative, Experiences, Perception, Involuntary admissions, Nigeria

## Abstract

**Background:**

People with mental health problems are more vulnerable to a broad range of coercive practices and human rights abuses. There is a global campaign to eliminate, or at the very least decrease, the use of coercion in mental health care. The use of coercion in psychiatric hospitals in developing countries is poorly documented. The primary aim of this study was to explore service users’ perceptions and experiences of coercion in psychiatric hospitals in Nigeria.

**Methods:**

Four focus group discussions were carried out among 30 service users on admission in two major psychiatric hospitals in Nigeria. The audio recordings were transcribed verbatim and then analyzed thematically with the aid of MAXQDA software.

**Results:**

The Focus group participants included 19 males and 11 females with a mean age of 34.67 ± 9.54. Schizophrenia was the most common diagnosis (40%, n = 12) and had a secondary school education (60%, n = 18). The focus group participants perceived coercion to be a necessary evil in severe cases but anti-therapeutic to their own recovery, an extension of stigma and a vicious cycle of abuse. The experience of involuntary admission revolved mainly around deception, maltreatment, and disdain. Participants in both study sites narrated experiences of being flogged for refusing medication. Mechanical restraint with chains was a common experience for reasons including refusing medications, to prevent absconding and in other cases, punitively. The use of chains was viewed by participants as dehumanizing and excruciatingly painful.

**Conclusion:**

The experiences of coercion by participants in this study confirm that human rights violations occur in large psychiatric hospitals and underscore the need for mental health services reform. The use of coercion in this context reflects agelong underinvestment in the mental health care system in the country and obsolete mental health legislation that does not protect the rights of people with mental health problems. The study findings highlight an urgent need to address issues of human rights violations in psychiatric hospitals in the country.

## Introduction

There is a global campaign to eliminate, or at the very least decrease, the use of coercion in mental health care. The negative impacts of coercive methods on both service users and care professionals who administer or witness them are becoming increasingly apparent [1, 2], while the evidence of their effectiveness and safety remains limited [3].

Coercion has been defined as “the act or practice of using force or threat to persuade a person to do something” [4]. It is a concept that applies to a broad spectrum of activities, ranging from mild acts such as persuasion on one end to the most oppressive acts of compulsion, such as the use of restrictive devices on the other end [5, 6]. Coercion in mental health care is broadly categorized as formal and informal [7]. While formal coercive practice describes tangible conspicuous acts of coercion such as compulsory admissions, seclusion (containing an individual in a secured room), physical (manual holding), mechanical (using restrictive devices like handcuffs) or chemical (pharmacological measures) restraint [4, 6], informal coercion is less obvious and exerts control in a more covert manner by using psychological and social mechanisms such as forceful persuasion or implied threat [5]. The study of coercion in mental health care is very relevant given that mental health problems could impair the ability to make decisions, making people with mental health problems particularly more vulnerable to a broad range of coercive practices and human rights abuses [8, 9]. The United Nations Convention on the Rights of Persons with Disabilities (UNCRPD), the most widely acclaimed international legislative instrument addressing the issue of coercion in mental health care [10], stresses the need for equality in legislation, policies, and practices that affect people with disabilities, including those with mental health conditions and psychosocial disabilities. It advocates a paradigm shift from the traditional paternalistic bio-medical approach to a human rights-based shared decision-making approach [11]. The use of coercion in the treatment of people with mental health problems is incompatible with the UNCPRD's principle of autonomy, which seeks to empower people with mental health problems to make their own treatment decisions.

The mental health system in Nigeria is severely under-resourced, with a psychiatrist-to-population ratio of roughly 1:1,000,000 and about 9.7 psychiatric nurses per 1,000,000 people [12]. Formal mental health care is accessed through psychiatric units in general hospitals/ secondary health care facilities, and mental hospitals (i.e., standalone psychiatric hospitals). Service users are typically severely ill and brought in by their families who are usually the sole carers of people with mental health problems in the country [13]. Although efforts are being made to incorporate mental health into existing primary health care [14, 15], just around 4% of the Nigerian health budget is dedicated to mental health, with over 90% of that expenditure going to running the country's psychiatric hospitals. According to the WHO-AIMS report in 2006, the country's human rights review commission does not monitor the actions of mental health facilities anywhere [16]. It was estimated that 51% of inpatients in psychiatric units in general hospitals/ secondary health care facilities and 64% of inpatients in the psychiatric institutions were involuntarily admitted. The report estimates the proportion of patients who have been restrained or secluded in the past year in both community and mental hospital setting to be 11–20%. It is not clear how these estimates were reached since there is no system for regular dissemination of patient and service data to the health ministry [16].

Nigeria is a signatory to many international human rights conventions including the African Charter on Human and Peoples’ Rights and the UNCRPD [17, 18]. Although the federal government has approved a national mental health policy, it is poorly implemented [19]. The Substance Use and Mental Health Bill 2019, which has gone through a public hearing, is expected to replace the previous colonial legislation and significantly enhance the rights of people with mental health and psychosocial disabilities in the country [20].

Coercive practices such as the use of chains, holding people with mental health problems hostage in cages, sheds, prayer camps and other forms of human rights violations have been documented in traditional healing centers and community settings in many low-and-middle-income countries (LMICs) including Nigeria [21–26], but there is little evidence on the use of coercion in psychiatric hospitals in these countries. There is a consensus on the need for more research on the subject matter in LMICs [27]. Since contextual factors are known to influence the practice and perception of coercion [26, 28], it is important to investigate the perception and experiences of coercion among service users in a family-centric, resource-limited setting [27, 29]. The study is the first qualitative investigation of service users' perceptions and experiences of coercive practices in Nigerian psychiatric hospitals. It’s a critical first step toward further research on the subject in the country, as well as the development of interventions and policies to address the problem.

## Methods

Focus group discussions were chosen as the appropriate method to obtain information about coercion among service users in the psychiatric hospital setting because focus groups are known to provide an opportunity for study participants to share difficult experiences within a potentially supportive environment and have been employed in previous studies regarding the same topic [30–32]. The dynamics of power between researchers and participants have been extensively studied. In their position as the seeker of information, researchers are usually considered to have more power. In many situations, such as when research is conducted among participants who have been diagnosed with a mental health condition, this power gradient may be exaggerated [33] and more so, when the participants are involuntarily admitted to a psychiatric institution. Not only do focus groups create multiple lines of communication among participants, but they also reduce the influence of the researcher, giving more dominance to participants and enabling them to share their ideas and opinions in the company of people who have similar experiences as them [34]. Study participants, most of whom were involuntarily admitted, may have felt coerced in an interview situation and consequently, may have adjusted their communication.

### Study setting

Two out of the eight major neuropsychiatric hospitals in the country, which cater to the mental health needs of about a quarter of the country’s population, were conveniently selected for the study. Study participants were recruited during their hospitalization in both study sites. Information about the study was disseminated by attending nurses and interested service users who met the eligibility criteria were co-opted into the study. The eligibility criteria were being at least 18 years old, admitted to the inpatient departments, certified to be stable by a psychiatrist, and able to give informed consent. A diagnosis of dementia was an exclusion criterion. The study included a total of 30 service users distributed into four different focus groups, a pair with eight people each and the other pair with seven people each. Participants with different sociodemographic characteristics (age and gender) were selected, and their distribution into each focus group was done in such a way as to favor the richness and heterogeneity of the discussion.

### Data collection

The focus groups were conducted between December 2021 and January 2022. Each discussion lasted between 45–60 min and was guided by a semi-structured discussion guide developed by the research team. The guide was not a rigid questionnaire, but rather served as a flexible framework for exploring study questions using open-ended questions to obtain unconditioned answers. The guide also served to homogenize the way the focus group discussions were conducted in each of the hospitals. The primary aim of the focus group discussions was to explore participants’ perceptions and experiences of formal coercive measures including involuntary admission and the use of restraints. The secondary aim was to explore suggestions on strategies to reduce the use of coercion in mental health care, which will be the subject of another paper by the research team.

The focus groups took place outside the wards, in halls within the hospitals to facilitate an unstructured atmosphere without pressure. The purpose of the study was carefully explained to participants at the beginning of the discussion, informed consent was obtained, and participants were given the option of leaving the discussion at any point when they felt distressed or unwilling to continue talking. They were assured that leaving the group or choosing not to participate would not affect their medical care in any way. To avoid the perception of a regulated environment, ground rules were made by consensus among participants and the moderators to facilitate the focus group discussion. Discussions were fluid and dynamic, with adequate interaction among participants. The focus group discussions were moderated by social workers in each hospital who had experience in qualitative research and underwent additional training for the study. They were responsible for facilitating and guiding the participation and discourse elaboration, while the content of the discussion was recorded in audio format. No relationship existed between the study participants and the social workers in all the cases, and the expectations of both parties were clarified at the beginning of each discussion. Participants were debriefed at the end of each focus group discussion. Given the diversity of languages spoken in Nigeria, with English being the *lingua franca* and the language of teaching at all levels of education, the focus group discussions were conducted in English. The study sites were anonymized to protect the psychiatric hospitals from being singled out for punitive measures due to the politicized nature of the findings given here.

### Data analysis

The audio recordings were transcribed verbatim and then analyzed thematically according to the recommendations of Braun and Clarke [35]. Transcripts were carefully read and reread by the lead investigator, then segments of text were coded, synthesized, and integrated into categories based on similarities of meaning. A general inductive approach was employed to code the data and coding continued until no new concepts emerged from the data. Coding consistency was checked by one of the co-investigators to ensure trustworthiness, and theme confirmation was reached after discussion among the lead investigator and two co-investigators. To be reflexive, the three authors (two of whom are psychiatrists working in the study sites) involved with the analysis tried to be self-aware and transparent about how our roles, perspectives and experiences may have influenced the analysis and interpretations [35, 36]. The two authors who are psychiatrists in the study sites may have been able to relate more to the experiences of the study participants and more likely have different interpretations of some actions deemed coercive since their job entailed prescribing coercive practices in certain cases. Due to the dual capacities as practitioners and investigators, there may have been a greater emphasis on some aspects than others, leading to the possible oversight of crucial themes and directions for understanding coercion in that setting. The dual role was discussed and reflected upon throughout the study, individually and with the lead investigator who is neither a mental health practitioner nor a service user. The analysis was performed with the aid of MAXQDA software [29]. Themes were summarized and interpreted using a contextualist approach where the characteristics of the study setting were used to understand the experiences of the participants [37]. Quotes were selected by the lead investigator to exemplify each theme. Ethical approval was obtained from the Research Ethics Committee of Nova Medical School, Nova University of Lisbon (100/2021/CEFCM) and the Ethics committee of each participating psychiatric hospital. Participants were required to sign a written informed consent before the Focus Group discussion and verbal consent was sought periodically until the end of the discussion.

## Results

There was a total of 19 male and 11 female participants. The mean age of participants was 34.67 (SD = 9.54) while the mean length of hospitalization was 36.53 ± 20.41 days. The most common diagnosis was schizophrenia and one-third of the participants (33.3%, n = 10) had a tertiary education while the rest were educated up to the secondary level. (Table 1).

**Table 1:** Participant socio-demographic characteristics (N = 30)

Characteristics	Frequency (n)	Percentage (%)
Gender		
Male	19	63.3
Female	11	36.7
Education*		
Primary	0	0.0
Secondary	18	60.0
Tertiary	10	33.3
Marital Status*		
Single	23	76.7
Married	3	10.3
Separated/Divorced/Widowed	3	10.3
Diagnosis		
Schizophrenia	12	40.0
Schizoaffective disorder	2	6.7
Bipolar disorder	3	10.0
Depression	3	10.0
Mental and behavioral disorder due to psychoactive substance	10	33.3
Status of Admission		
Voluntary	13	43.3
Involuntary	17	56.7
Number of previous admissions		
0	20	60.7
1	6	20.0
2	1	3.3
3	2	6.7
> 3	1	3.3

^*^2 participants did not indicate their educational level

^*^1 participant did not indicate their marital status

### Perception of coercion

#### Coercion as a necessity

Many participants believed that coercion was a ‘necessary evil’ for patients who lacked insight, were stubborn, or severely ill. They, however, suggested that it should be left as a last resort after other options like negotiation and dialogue have been explored. Participants with a diagnosis of mental health and behavioural disorder due to psychoactive substance (MBDPS) were particularly indignant about being coerced and felt that they should not be subjected to coercive practices since they had insight.

“....Coercion is something that is necessary. That is a must, based on the mental illness ... because at the point where the illness begins, ... the patient in question, may not necessarily understand,... and may not even want to come to dialogue. Because at that point in time..., there's restlessness .... at that point in time, there's apprehension... depending on the ailment. And so coercion is a must. it's a must that must be used.”(FGD1, male participant with Bipolar Disorder)

“There are some patients that truly need to be forced. They're mentally insane [sic], yes. Psychoses, real ones....the people that are diagnosed with drugs has a better understanding than those that are real psychoses.” (FGD2, male with MBDPS)

#### Coercion as a control tool

Most participants agreed that acquiescence to requests made by family and healthcare professionals precluded the use of coercion, while resistance and arguments led to the use of stiffer coercive measures.

“If you begin to, you know, argue with the people that brought you here or with the medical personnel that is attending to you, it would be difficult, they will coerce you, but if you agree if you obey, if you begin to bear all whatever they say, or even make some little argument before you give in, they will not coerce. The only coercion will be just that of persuasion with words, not by whipping or chaining you.”(FGD4, male participant with schizophrenia )

“When you willingly adhere to instructions. Like for instance, they tell you do this, you do it, you do that.... you're not giving them any reason to forcefully coerce you.” (FGD1, female participant with MBDPS)

#### Anti-therapeutic and traumatic

Many participants felt that coercion was not an effective way of managing people with mental health problems, but rather aggravated the distress that they felt in times of crisis. It made them feel worse about the diagnosis, and receiving care from the same people who subjected them to various coercive practices was traumatic.

“To me, is abnormal because it deals with mental health. Okay. Chaining, injections and all that, it affects psychologically. So, it's not proper.” (FGD1, male with MBDPS)

“Still being in that same environment, looking at the same people that did that thing, perhaps those ones may not be related to the person in the hospital but then, it can never go well, even in terms of...receiving health care from them, medications here and there, the person will never be the same, will never be happy because he has been...abused, bullied.”(FGD2, male with MBDPS)

#### Coercion as an extension of stigma towards people with mental problems

A few participants were able to link coercion with the discrimination they experienced because of their mental health condition. Coercion was a form of stigmatisation, and in other cases, a response to their protests against stigma and discrimination.

“So, it’s an injustice that some people are being stigmatised and unwelcome in their very home because of what they believe in ….So, you decide because you have the upper hand in the family to take care of me, give me food three times a day, to call in security people from even this facility to come and take me away for admission.” (FGD3, male participant with depression)

“I have pains all over but my family is trying to cover up with something like I shut the doors, I didn't want to open the doors for anybody. Why I didn’t want to open the doors for anybody is because of.... they said some parable;...if somebody call you somebody, you call that person somebody. If they don't call you person, don't call the person ‘person’. Who address you as a human, you also address that person as a human. That’s why I shut the door because my family never addressed me as a normal human.” (FGD2, male participant with MBDPS)

#### Coercion as a vicious cycle

Some participants pointed out how coercion evoked aggressive behaviours and made service users want to abscond, and these behaviours, in turn, led to stiffer coercive measures, leading to an unending cycle of aggression and coercion.

“Because the way you approach someone that's how the person will retaliate to you. that's true. The way you approach someone is how that person will retaliate to you.” (FGD2, male participant with MBDPS)

“...when I notice coercion practises on me. I feel so aggressive [sic]. So, I feel so aggressive because...had it been they used peacefully, it will be better for me than to use coercive issue. (FGD3, female participant with schizophrenia)

### Experiences of involuntary admission

#### Deceived and kept in the dark

Many participants narrated how they were tricked into going to the psychiatric hopitals and then admitted against their will. They complained about being taken unawares and, in some cases, it seemed to them like they were being kidnapped.

“You cannot just come into deceiving, you know. In my own case I was deceived. I was, they told me I'm going for my business work, but on seeing this sign board, I now notice that they have lied to me. So that’s what I'm saying that the whole system is illegal. Because if they had told we are going to psychiatric hospital for evaluation, I will know and that's what I thought. They told me that I'm coming for work that they will pay me after my work, with that in mind, I started coming.” (FGD4, female participant with schizophrenia )

“Before I could say Jack or James, they took me. I was with my friends, And I was praying, this is what I have been hearing of. Assassins, they can kidnap someone and get to the expressway and just finish up the person but to me, I said well, I'm old enough, if I die, I die. No problem, but all I know is that I need somebody around the environment to know that I'm gone. Somehow, we started the journey and I was asking the gentleman beside me, where are you taking me to. He didn’t mention and I became very scared.” (FGD2, male participant with MBDPS)

#### Admitted for the wrong reasons

Most participants felt their admission was unnecessary, and they were brought by their families for punitive reasons. The threshold of tolerance for misdemeanours seemed lower for people with mental health problems, as disagreements could lead to admissions.

“I have had experiences of being called from home by security men. you understand? and I've had experiences of involuntary admissions because someone believe that you are not in good terms with the person makes the person to feel like he's obliged because you're under his or her care to drive you to this mental health care service so that they will deal with you. Because they themself at home could not deal with you.”(FGD3, male participant with depression)

“The second time they brought me here, it was just a fight. It was just a fight with somebody, they did not even investigate the cause of the fight, they just brought me and bundled me back into this place.... so, if not that I have more understanding, I would have overreacted... How can someone beat me and molested me like this? And the next thing for you to do is to take me to the hospital, psychiatric hospital.”(FGD1, male participant with Bipolar disorder)

#### Maltreatment and suffering

For most participants, the process of being admitted was a harrowing experience. They felt dehumanised and manhandled in the process of being brought to the hospital to be admitted involuntarily. Several participants reported being injured by the ropes and chains used to restrict movement while being transported to the hospitals.

“.... My experience with them is that … they were supposed to counsel us, ... they don't have conscience, they didn't us treat us like human. They did not treat me like as if I'm a human being, despite my complain in the office there. Cuz I was against coming here. And I'm coming here for the second time for no good reason, in short, I talked throughout that day, almost to the point of killing myself because there was weapon with me because I was frustrated to that extent.” (FGD1, female participant with schizophrenia)

“Like me I was being injured, tied with a rope and brought me here. Even my leg. You can see my leg. I have pain on my leg, even in my hands. I have pains all over.”(FGD2, male participant with MBDPS)

“Like me o, they tie me o, they tie me like Jesus Christ and sedated me [sic]. it's very very bad.” (FGD1, male participant with schizophrenia)

#### Ignored and dismissed

Most participants affirmed that they had opinions at the time of admission and expressed strong displeasure at not being listened to during the admission process. Everything they said was dismissed as gibberish or as a result of being under the influence of drugs for service users with a history of substance use. Others noted that nobody bothered to meet the basic needs of service users like hunger and thirst at the time of admission.

“So, I'm coming for the second time now for no good reason despite my complain, all my shouting, nobody listened to me, it was as if I was just like a fool that doesn't know what she is saying. At least they should have given me some listening ear, counsel me and see if I can be admitted or not.” (FGD1, female participant with schizophrenia)

“They cannot let us speak our mind, cuz we have something to say. Yes. (All participants agree in unison) That period, we have something to say but they don't allow us to speak our mind, they will bundle us, tie us. During the time you are forcing that person to chain him or in order to tie him or bring him down to this very place, you didn't know if he ate or not, You didn't know if he is sick, or whether he ate, or whether he have a problem, that kind of person, he can die.” (FGD1, male participant with schizophrenia)

### Experience of Chemical Restraint

#### Undesirable effects

Many participants (43.3%, n = 13) complained that the chemical restraints had negative effects on them, from sleeping for prolonged periods, inability to walk because they were injected into their thighs, and dyskinesia. Many participants described the process of administering the chemical restraint as humiliating because their clothes were forcibly removed to administer the injections.

“E get some kind of injection, you give some patients here, the body will change automatic, some will start turning their eye, some will start shaking their body and I want the hospital look into... it.” (FGD3, male participant with MBDPS)

“I was injected with sleeping injection, so I slept off that night. When I woke up in the morning, they've injected my both laps. I can't even walk for like a week and some days.” (FGD3, male participant with MBDPS)

“So, I was held down, pinned down and my... covering was physically and manually pulled down off my waist and the syringe was inserted into my body and alongside I had some bruises on my fist. I think it’s a very bad experience.” (FGD2, male participant with MBDPS)

“...and when they injected me, I slept immediately and those that were with me said that is for two consecutive days that I didn't wake up. So that's the effect of the drugs.” (FGD4, male participant with schizophrenia)

#### Safety concerns

Some participants were concerned about the saftey of injections for chemical restraint, and felt injections should be used in severe cases only.

“You know injection pass through the vein now. So, injection should be given to somebody depending on the case of that person, not somebody have issue that ordinary tablets can cure, ...You carry injection and be giving to the person.” (FGD3, Female participant with schizophrenia)

### Experience of ECT without consent

None of the study participants had been subjected to ECT without their consent. One participant however had seen a ward mate who had undergone ECT. It is unclear whether the patient’s consent was sought before the procedure.

### Experience of mechanical restraint

#### Chained like an animal

The use of chains for mechanical restraints was a reoccuring theme across all the questions addressed in the focus group discussions in both study sites (40%, n = 12). It was a practice affirmed to be wrong, dehumanizing, abnormal, humiliating and excruciatingly painful by the participants. It was mostly used in response to resistance to chemical restraint and to prevent absconding, however, the process was also identified as a punitive measure in some cases. They also discussed the deleterious effects of the chains ranging from physical injuries to bladder infection as a result of restriction of movement.

“My experiences are basically use of chain. The first day they admitted me at *Study site 1*, they forced me to pull off my shirt, I refused. They dragged me and they chained me. The pain was so excruciating. That's my own experience of physical abuse.”(FGD2, male participant with bipolar disorder)

“No, nobody supposed to be treated as an animal. For you to be forced to have a chemical, to be injected, or to be chained is not normal. I don't think it’s normal.”(FGD2, male participant with MBDPS)

“Ok. I was chained in *Study site 1* for reason of leaving the hospital. In case I want to leave the hospital voluntarily. So, I cannot leave, so they had to chain my leg to the bed all night, I couldn't even use the washroom. That was a bad experience. I think I have bladder infection.” (FGD2, male participant with schizophrenia)

“and there was one other time..., they was chaining somebody. I was now trying to tell the guy please o, the chain is too tight on that guy. The person that was using the chain came and used the chain to whip me, ...and restrict me to my bed... I felt very bad. Because I was telling him that the chain is too tight on that person's leg, he used the chain on me. So... they themselves, they abuse the process...those people that are using... both the chemicals and chains, they abuse the process and it's bad. I feel it’s very bad.” (FGD1, male participant with bipolar disorder)

“I was trying to desist them from,... giving me injection and when they wanted to give me injection, I was trying to resist them, they...chained me and they keep me out there, at the pole there... that place where they're doing ward round.”(FGD4, male participant with schizophrenia)

#### Whipping

Some participants (16.6%, n = 5) in both study sites recounted experiences of physical abuse in the institutions, most of which occurred as a result of rejecting medications or disobedience to stipulated rules.

“That was the most craziest day of my admission, that was the day I tore my shirt to pieces because the way I was treated that day wasn't right at all. Cuz I was following all the rules and regulations they gave me but still yet, I was beaten that day, yes, I was beaten and clamped to the bed and chained...they chained my two legs and that day was the worst day of my admission ever.” (FGD2, male participant with MBDPS)

“What encourage coercion or chain is disobedience and refusing to be injected. That will make them to flog us or chain us.” (FGD 4, male participant with MDPS)”

“…. they will force you and some they will even flog you cane when they are giving you the injection.” (FGD3, male participant with MBDPS)

## Discussion

The broad categories presented in the paper are based on the questions in the focus group discussion guide and the themes that emerged after an inductive analysis [38] of the data obtained. The focus group discussions appeared to be an avenue for the participants to vent out concerns and frustrations about the system in an unencumbered way.

Although the general feeling about the use of coercion was negative, it was viewed as indispensable by the majority of the participants. They identified two types of patients in the wards: those who deserved and would indeed ‘benefit’ from being coerced because they lacked insight and those like themselves who did not deserve to be coerced because they knew what they were doing. Participants in the study appeared to stigmatise those they thought had more serious mental health problems, which might explain why they endorsed coercive practices for this group of patients but not for themselves. It was also generally agreed that other options like dialogue and negotiations were to be explored before the use of coercion as a last resort. The view that coercion is a necessary evil to prevent greater evil is in keeping with previous studies among service users and mental health care staff [39–41].

The procedure for involuntary admission in Nigeria differs from elsewhere, as there are no regulatory mechanisms to oversee the process of involuntary admissions. Like many LMICs, there is little or no social welfare service in the country [23, 42]. Family and friends are entirely responsible for the care and support of people with mental health problems. The decision on whether to admit a person to psychiatric institutions is wholly dependent on the family. It is also important to clarify that the cost of admission ( which can be as high as five times the country’s minimum wage) is borne by the family [43], and is a major determinant of the decision to seek help from formal health care facilities in the first instance. This implies that only people with mental health problems whose families can afford the cost of treatment can be admitted involuntarily. To avoid struggle, absconding, and the additional cost of involving the police, many families resort to deception to take patients to psychiatric facilities. Many participants had this experience and expressed their resentment at being lied to. One participant highlighted that an admission based on deception and patients’ perceived lack of need for treatment significantly reduced the chances of benefiting from treatment during the admission. Theodoridou and colleagues showed in their study that perceived coercion among service users evokes negative feelings that hinder the development of positive therapeutic relationships with care providers [44]. However, the long-term impact of these negative feelings on the therapeutic outcomes remains unclear. The fact that they were not listened to and that their opinions were dismissed was a significant issue highlighted by one of the participants, supported by every other participant in the focus group discussion. As they rightly pointed out, this could be dangerous since pre-existing conditions could be made worse by coercive measures. This disregard for what people with mental health problems have to say has been documented as a core experience among psychiatric patients [45] and might be a contributing factor to the large mortality gap among this group [46]. The study participants felt dehumanized during admission, corroborating similar findings from previous studies conducted in Australia [31] and the United Kingdom [40]. Dehumanization has been consistently linked to some of the worst interpersonal behaviors like negligence, aggression and support for torture [47–49]. Dehumanization of people with mental health problems is closely associated with stigmatization and has been shown to be directly related to harsher responses to moral dilemmas involving patient care [50]. Interventions that reduce stigmatization and dehumanization among mental health professionals could be potentially helpful in reducing coercive practices.

Regarding chemical restraints, the participants affirmed that they were used to calm them down, but did not like the prolonged effects. It is possible that the medications were administered to treat symptoms rather than as chemical restraints, with sedation as a convenient side effect. More participants in one of the study sites than the other complained of the prolonged effect of the chemical restraints, which made them sleep for days and unable to walk. On enquiry, it was explained that there was a shortage of orderlies to guard the hospital premises or chase patients who tried to escape, and so chemical restraints were used to immobilise the patients. Understaffing was linked to verbal abuse of patients in psychiatric institutions in a recent WHO QualityRights study carried out in four West African countries [51]. The need to increase resources for mental health care in the country cannot be overemphasized.

The use of chains for mechanical restraints was a recurring theme across all questions addressed in the four focus group discussions held in this study. The use of chains was the most traumatic and dehumanising form of coercion for most participants. When they were not flogged with the chains, they were used in the process of being involuntarily admitted, usually preceding the use of chemical restraints, and for punitive purposes. Using coercive measures for punitive purposes was also reported in the recent QualityRights study [51]. The use of chains for the restriction of movement in people with mental health problems is widely known to occur in homes, prayer houses, and traditional healing homes in the country [52]. The study participants were right to be perplexed that chains were also used in psychiatric hospitals by mental health care professionals who were supposed to understand the psychological impact of using chains better than anyone else. There have been media campaigns on the need to end coercion in non-formal settings in Nigeria [52, 53], but not in hospital settings. Apart from the physical and psychological trauma caused by chains, other unanticipated consequences, such as the deaths of several admitted patients who were unable to escape a fire outbreak because of the chains, have been reported in India [54]. There is an urgent need to address these significant human rights violations at various levels, including legislation related to people with mental and psychosocial problems. This implies more investment in the mental health care system, development of community-based mental health services, routine training of mental health workers, regulatory mechanisms to check human rights abuses and the integration of mental health care in primary health care to make it more accessible and less stigmatising.

The relatively high prevalence of coercive practices in high-income countries with more resources for mental health care indicates that these resources do not always equate to high-quality services based on human rights [55], unless specific efforts are made to reduce coercion and promote the rights of service users. So investment of more resources in mental health care should go hand in hand with a human rights oriented service reform. Shared-decision making instead of paternalism is useful in managing the power dynamics between patients and clinicians [56]. The recently launched WHO mental health report notes that to prevent human rights violations in mental health services, a mix of strategies are needed including the scaling up of recovery oriented community based services, monitoring and evaluating mental health services and actively involving people with lived experience of mental health conditions in decision-making processes [57]. The WHO QualityRights initiative which is based on the CRPD principles, seeks to improve psychiatric services and promote the rights of persons with mental health problems. The QualityRights toolkit was developed for use in low- and middle-income countries and provides practical knowledge and tools for assessing and enhancing quality and human rights standards in mental health and social care facilities [58]. Finally, training of mental health professionals on human rights and alternative strategies to coercion is a key strategy that has been shown to be most effective in reducing coercion in mental health services [59].

### Study limitations

Only two psychiatric hospitals were included in this study, and it is unclear if the practices are similar in other hospitals across the country. Verbal abuse and other informal coercive measures were not explored in this study and should be investigated in future research.As with all qualitative studies, the current study suffers from the limitation of extrapolation beyond the context of the study. However, the objective of the study was to explore and describe the experiences of the study participants, and not to generalize study findings [60]. Although this study was done in standalone psychiatric hospitals in Nigeria, the results are probably comparable to psychiatric units in general hospitals. If there are any disparities, they may be related to the severity of cases seen in the two settings, with patients in standalone hospitals typically receiving patients with more severe mental health problems. The similarity points to the cultural undertone of Nigeria’s use of coercion in mental health care. To fully comprehend this, additional research that include the general hospital and community settings are required. Other potential limitations include the exclusion of ‘unstable patients’ whose experiences may significantly differ from those of included patients and who are more likely to be victims of coercive practices. None of the study participants had been subjected to ECT without their consent. ECT is usually a last resort for resistant cases, and patients with severe conditions that may have necessitated the use of ECT were not selected for the study.

## Conclusion

The use of coercion was viewed by the study’s participants as a necessary evil for people with severe mental health problems who lacked insight. However, it was also highlighted as being inefficient in the long run. The experiences of coercion by participants in this study confirm that human rights violations occur in large psychiatric hospitals and underscore the need for inpatient service reform and the development of recovery oriented community-based mental health services in the country. The use of coercion in this context reflects agelong underinvestment in the mental health care system in the country and obsolete mental health legislation that does not protect the rights of people with mental health problems. The study findings highlight an urgent need to address issues of human rights violations in psychiatric hospitals in the country.

## Data Availability

The data analyzed during the current study are available from the corresponding author on reasonable request.
